# Population size estimation for quality control of ChIP-Seq datasets

**DOI:** 10.1371/journal.pone.0221760

**Published:** 2019-08-29

**Authors:** Semyon K. Kolmykov, Yury V. Kondrakhin, Ivan S. Yevshin, Ruslan N. Sharipov, Anna S. Ryabova, Fedor A. Kolpakov

**Affiliations:** 1 BIOSOFT.RU, LLC, Novosibirsk, Russian Federation; 2 Institute of Computational Technologies SB RAS, Novosibirsk, Russian Federation; 3 Institute of Cytology and Genetics SB RAS, Novosibirsk, Russian Federation; 4 Novosibirsk State University, Novosibirsk, Russian Federation; Auburn University - Harrison School of Pharmacy, UNITED STATES

## Abstract

Chromatin immunoprecipitation followed by sequencing, i.e. ChIP-Seq, is a widely used experimental technology for the identification of functional protein-DNA interactions. Nowadays, such databases as ENCODE, GTRD, ChIP-Atlas and ReMap systematically collect and annotate a large number of ChIP-Seq datasets. Comprehensive control of dataset quality is currently indispensable to select the most reliable data for further analysis. In addition to existing quality control metrics, we have developed two novel metrics that allow to control false positives and false negatives in ChIP-Seq datasets. For this purpose, we have adapted well-known population size estimate for determination of unknown number of genuine transcription factor binding regions. Determination of the proposed metrics was based on overlapping distinct binding sites derived from processing one ChIP-Seq experiment by different peak callers. Moreover, the metrics also can be useful for assessing quality of datasets obtained from processing distinct ChIP-Seq experiments by a given peak caller. We also have shown that these metrics appear to be useful not only for dataset selection but also for comparison of peak callers and identification of site motifs based on ChIP-Seq datasets. The developed algorithm for determination of the false positive control metric and false negative control metric for ChIP-Seq datasets was implemented as a plugin for a BioUML platform: https://ict.biouml.org/bioumlweb/chipseq_analysis.html.

## Introduction

Understanding the basic mechanisms of transcription regulation remains to be the great challenge in modern biology. Regulation of transcription is a complex process in which transcription factors (TFs) play the key role. As a rule, TFs recognize and bind with corresponding TF binding sites (TFBSs) in the genome. The *in silico* recognition of those TFBSs in whole genomes has been staying one of the most complex problems in bioinformatics. Nowadays, chromatin immunoprecipitation followed by sequencing (ChIP-Seq) is a widely used experimental technology for the identification of TF binding regions (TFBRs) containing TFBSs. For now, tens of thousands of ChIP-Seq experiments have been conducted. It is reasonable to assume that this number will increase rapidly year by year.

By now, several databases such as ENCODE [1], GTRD [2], ChIP-Atlas [3], and ReMap [4] have been created. New distinct datasets have been systematically collected, annotated, and uniformly processed there, including data on TFBRs obtained by application of different peak callers to primary ChIP-Seq data. It is naturally to assume that increasing number of collected datasets demands not manual, like before, but automatized assessment of quality to simplify selection of proper datasets for further analysis. Currently, the common practice to assess the quality of ChIP-Seq datasets is to apply well-known quality metrics developed within the ENCODE project. For instance, the metrics such as NRF (Non-redundancy Fraction), PBC1, PBC2 (PCR Bottlenecking Coefficient 1 and 2), NSC (Normalised Strand Cross-correlation coefficient), and RSC (Relative Strand Cross-correlation coefficient) are applied to measure the quality of the read alignments to individual genomes [5]. To estimate directly the quality of ChIP-Seq datasets produced by distinct peak callers, the FRiP (Fraction of Reads in Peaks) metrics is commonly used [5].

Up to date, at least three databases such as ENCODE, GTRD and ReMap assess all their ChIP-Seq datasets with the help of the mentioned metrics. However, it seems likely that such issue as quality control of ChIP-Seq datasets has been incompletely addressed. In particular, existing quality metrics do not allow to control the false positive (FP) and false negative (FN) rates in datasets generated by distinct peak callers. The main goal of our study was to develop two novel quality control metrics, False Positive Control Metrics (FPCM) and False Negative Control Metrics (FNCM), which allowed to control FP and FN rates of peak callers. For this purpose, we used methods for population size estimation in order to estimate unknown number of genuine TFBRs.

Basically, estimation of population size is intensively utilized in many fields of knowledge, including ecological sciences [6], medicine [7] and social sciences [8]. In general, a number of capture-recapture models tend to be applied in a variety of applications including estimation of population size. However, these models have not been applied for analyses of ChIP-Seq datasets. Certainly, the main aim of the developed metrics is to serve as a guide for selection of more reliable datasets as well as for creation of their modified versions. We also have shown that the proposed metrics appeared to be useful for other applications such as comparison of peak callers or prediction of TFBSs within TFBRs.

In general, accurate identification of TFBSs is still a big challenge in bioinformatics. Currently, position weight matrix (PWM) approach is one of the most common and widely used for computational identification of TFBSs. A number of methods for prediction of the putative TFBSs has been developed within this approach. In particular, MATCH [9], MEME [10], and HOCOMOCO weight matrix model [11] are among them. There are several repositories that accumulate matrices for representation of TFBSs. In particular, HOCOMOCO [11], JASPAR [12] and UniPROBE [13].

Currently, more than 30 peak calling algorithms have already been published to derive TFBRs datasets from aligned ChIP-Seq data [14]. At present, various comparative analyses of such algorithms have already been carried out. One of the first comparative analyses was published in 2009 [15]. However, undoubtedly, the best algorithm for peak calling has not been found so far. As a rule, those comparisons were usually made on a small number of datasets while using various metrics and comparison criteria. Consequently, some comparative analyses led to conflicting evaluations. For example, in three analyses the conflicting conclusions were made for algorithms such as MACS, SICER and F-Seq [16, 17, 18]. The current state of the art unambiguously indicates the high demand to develop more sophisticated metrics and comparison criteria, as well as to create a single and representative test dataset that can be used in further comparative analyses.

## Materials and methods

### Algorithm for determination of FPCM and FNCM

Let D denote meta-set D = {D_1_, …,D_k_} consisting of k datasets of TFBRs D_i_, i = 1, …,k. We considered two following dual settings. In the first case, D_1_, …,D_k_ are datasets of TFBRs obtained by independent application of k distinct peak callers to the same ChIP-Seq set of reads aligned to the reference genome. In particular, we considered the following k = 4 peak callers available in GTRD: GEM [19], MACS [20], PICS [21], and SISSRs [22]. In the second case, a meta-set contains TFBRs datasets obtained by application of single peak caller to the distinct ChIP-Seq sets of reads when the same TF was studied in different ChIP-Seq experiments. We developed our FPCM and FNCM metrics to assess the quality of individual datasets D_i_, i = 1, …,k as well as the whole meta-set D.

**Fig 1:**
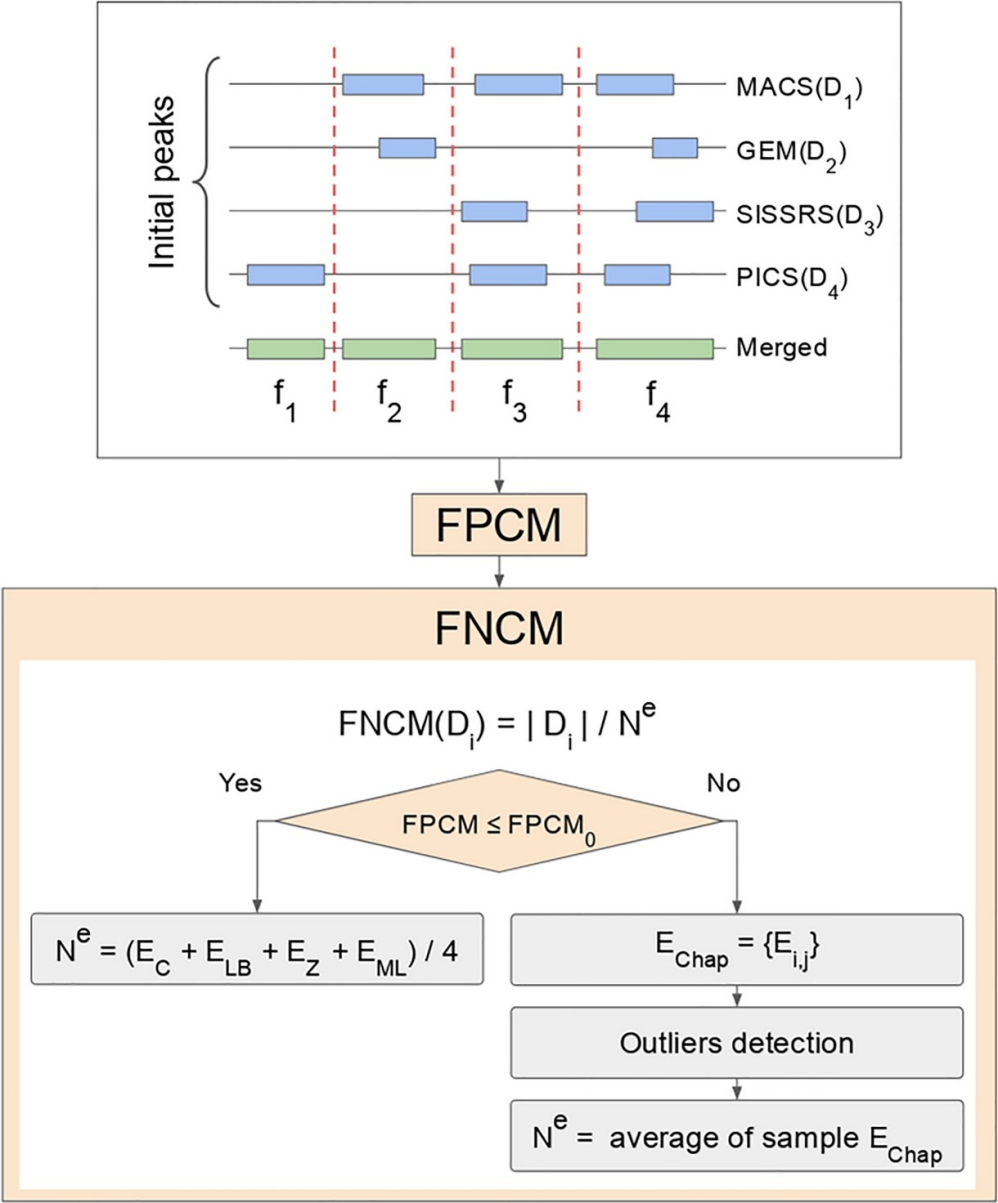
The workflow of algorithm for determination of FPCM and FNCMs.

To derive FPCM and FNCM, we initially merged all TFBRs available in meta-set D, see Fig 1. After that, the pivotal frequencies f_1_, …,f_k_ were counted on the basis of all merged TFBRs where f_i_ was defined as the number of merged TFBRs that were composed by exactly i TFBRs from meta-set D. In particular, f_1_ + …+ f_k_ = n where n denotes the number of all merged TFBRs. On the one hand, f_k_ is the frequency of those merged TFBRs that contained initial TFBRs from each D_1_, …,D_k_. On the other hand, f_1_ is the number of so-called orphans, i.e. such TFBRs that did not overlap with other initial TFBRs.

To control FP rate, we defined FPCM under the natural assumption that almost all FP TFBRs must be orphans, i.e. not confirmed by other TFBRs. Additionally, we assumed that unknown number of genuine TFBRs is a random variable with Poisson distribution. Under these assumptions, FPCM was defined as the ratio of the observed number of orphans f_1_ to the unknown number of genuine orphans f_1_^gen^, i.e.

$$FPCM=\frac{f_{1}}{f_{1}^{gen}}.$$ (1)

The estimate f_1_^e^ of the unknown f_1_^gen^ was derived, in turn, as solution of the system of three equations

$$p_{1}=\lambda e^{-\lambda },$$ (2)

$$p_{2}=\lambda^{2}\frac{e^{-\lambda }}{2},$$ (3)

$$p_{2}=\lambda^{3}\frac{e^{-\lambda }}{6},$$ (4)

where λ is unknown parameter of Poisson distribution, and pi is a probability that randomly chosen merged TFBR is composed by exactly i initial TFBRs. As a result, the final version of FPCM is expressed as

$$FPCM=\frac{f_{1}}{f_{1}^{e}},$$ (5)

$$f_{1}^{e}=2\frac{f_{2}^{2}}{3f_{3}},$$ (6)

where f_1_^e^ is the expected number of orphans.

The detailed derivation of FPCM is as follows. According to formulas for p_1_ and p_2_, the ratio p_2_ / p_1_ is equal to λ / 2, hence

$$\lambda =2\frac{p_{2}}{p_{1}}.$$ (7)

According to formulas for p_1_ and p_3_, the ratio p_3_ / p_1_ is equal to λ^2^ / 6, hence

$$p_{1}=6\frac{p_{3}}{\lambda^{2}}.$$ (8)

According to formulas (7) and (8), p_1_ can be estimated as p_1_^e^ where p_1_^e^ = 2 p_2_^2^ / (3 p_3_). Finally, the formula (5) for FPCM = p_1_ / p_1_^e^ was obtained with the help of replacement of probabilities p_1_, p_2_, and p_3_ by frequencies f_1_, f_2_, and f_3_ respectively.

In general, we can assume that each set of orphans may consist of True Positive Orphans (TPOs) and False Positive Orphans (FPOs), i.e. number of orphans (f_1_), can be expressed as

$$f_{1}=(number\;of\;TPOs)+(number\;of\;FPOs).$$ (9)

The number of TPOs was estimated as f_1_^e^ with the help of Poisson distribution. Therefore the proportion of FPOs (say, p_false_) can be estimated as

$$p_{false}=\frac{f_{1}-f_{1}^{e}}{f_{1}}=1-\frac{1}{FPCM}$$ (10)

and FPCM can be re-expressed as FPCM = 1 / (1—p_false_). If Poisson distribution is not contaminated then f_1_ and f_1_^e^ have to approximately coincide. In this case p_false_ has to be negligible and FPCM has to be close to 1. However, if Poisson assumption is seriously violated then proportion p_false_ takes large values and FPCM considerably exceeds 1. For example, if p_false_ = 1/2, 2/3 or 0.98, then FPCM takes the values 2.0, 3.0 and 50.0 correspondingly. In other words, If FPCM takes the values 2.0 or 3.0 then a half or more than a half orphans are FPOs.

Basically, FPCM can not only assess the quality of datasets, but also can recommend to modify them to improve their quality, if necessary. Thus, if FPCM exceeds a prespecified threshold, such as FPCM_0_ = 2 or 3, then FPCM recommends to modify dataset by removing orphans because in these cases, at least, a half (p_false_ = 1/2) or a majority (p_false_ = 2/3) of them are falsely generated by peak callers.

To control FN rates in D_1_, …,D_k_ datasets, we defined FNCMs for each of them. Thus, FNCM was defined for every Di as the ratio of the observed number of TFBRs in Di to the unknown number of genuine TFBRs, say, N^gen^, i.e.

$$FNCM(D_{i})=\frac{\left|D_{i}\right|}{N^{gen}},$$ (11)

where | D_i_ | denotes the size of the D_i_ dataset. If FPCM is less than the prespecified threshold (FPCM_0_), then it is not necessary to modify initial datasets, and N^gen^ is estimated as the average of the four distinct published estimates E_C_, E_LB_, E_Z_, and E_ML_ of the N^gen^, i.e.

$$FNCM(D_{i})=\frac{\left|D_{i}\right|}{N_{1}^{e}},$$ (12)

$$N_{1}^{e}=\frac{\left(E_{C}+E_{LB}+E_{Z}+E_{ML}\right)}{4},$$ (13)

where E_C_ is Chao’s estimate [23], E_LB_ is Lanumteang-Bohling’s estimate [24], E_Z_ is Zelterman’s estimate [25] and E_ML_ is maximum likelihood estimate [26]. The explicit forms of these estimates are as follow:

$$E_{C}=n+\frac{f_{1}^{2}}{2f_{2}},$$ (14)

$$E_{LB}=n+\frac{3f_{1}^{3}f_{3}}{4f_{2}^{3}},$$ (15)

$$E_{Z}=\frac{n}{1-exp(\frac{{-2f}_{2}}{f_{1}})},$$ (16)

$$E_{ML}=\frac{n}{1-exp(-\lambda^{*})},$$ (17)

where λ* is calculated numerically by maximization of log-likelihood function L(λ) of zero-truncated Poisson distribution,

$$L(\lambda )=constant+\log(\lambda )\sum_{i=1}^{k}(i*f_{i})-n\;\log\left(e^{\lambda }-1\right).$$ (18)

If FPCM exceeds the prespecified threshold FPCM_0_, then it is necessary to modify initial datasets by removing orphans. In this case the estimates E_C_, E_LB_, E_Z_, and E_ML_ are degenerated because f_1_ is vanished (f_1_ = 0) due to discarding orphans. To obtain new estimate of unknown number of genuine TFBRs N^gen^, we considered all k(k-1)/2 distinct pairs (D_i_, D_j_)_i<j_ and calculated for each pair (D_i_, D_j_) the Chapman’s estimate [27] E_i,j_ by the formula

$$E_{i,j}=\frac{\left(|D_{i}|+1)(|D_{j}|+1\right)}{|D_{i}\cap D_{j}|+1}-1$$ (19)

Then we checked for outliers in the obtained sample E_Chap_ = {E_i,j_} and discarded the detected outliers. An arbitrary element X in sample is classified as outlier, if the following inequality holds:

$$|\left(X-X_{0}\right)|>3\sigma ,$$ (20)

where X_0_ and σ are mean value and standard deviation when X is temporary removed from the sample E_Chap_. Finally, N^gen^ is estimated as the average of sample E_Chap_ and FNCM(D_i_) is expressed as

$$FNCM(D_{i})=\frac{\left|D_{i}\right|}{N_{2}^{e}},$$ (21)

$$N_{2}^{e}=average\;of\;sample\;E_{Chap}.$$ (22)

FNCM varies in the range [0.0; 1.0]. The closer the value of FNCM to 1.0, the lower is the rate of FNs, while values closer to 0.0 indicate that high number of genuine TFBRs was overlooked.

## Results and discussion

### FPCM and FNCM: Guidelines for dataset selection and modification

To get the first idea about some particularities of FNCM and FPCM, we applied independently the proposed algorithm for their calculation to two meta-sets derived from the GTRD database developed by our team [2]. Meta-sets PEAKS035099 and PEAKS039626 contained TFBRs of TF CTCF and were generated by the following peak callers in GTRD: GEM, MACS, PICS, and SISSRs. According to Fig 1, we merged initially four individual datasets PEAKS035099 generated separately by those four peak callers. Then, the pivotal frequencies f_i_ were computed on the base of 44699 merged TFBRs: f_1_ = 5534, f_2_ = 4542, f_3_ = 2482, and f_4_ = 32141. The value 0.998 of FPCM was calculated by formula (5). One can conclude that there are almost no FPs among TFBRs because FPCM was approximately equal to 1.0. In other words, the observed number of orphans was approximately equal to the expected one. To estimate the total number of genuine binding regions N_1_^e^ in this case, it is sufficient to calculate the arithmetic mean of four estimates:

$$E_{C},=48070,\;E_{LB}=48066,\;E_{Z}=55437,\;and\;E_{ML}=46525.$$

Finally, the estimated number N_1_^e^ = 49525 of TFBRs was used to compute FNCMs for all four individual datasets PEAKS035099. Table 1 contains these values.

**Table 1:** FPCMs and FNCMs for several meta-sets of TFBRs.

Meta-set	TF (TF-class)	FPCM	FNCM			
			GEM	MACS	PICS	SISSRs
PEAKS035099	CTCF (2.3.3.50.1)	0.998	0.776	0.874	0.689	0.702
PEAKS039626	CTCF (2.3.3.50.1)	24.901	0.881	0.929	0.767	0.899
PEAKS033754	CTCF (2.3.3.50.1)	0.782	0.871	0.861	0.483	0.141
PEAKS033837	GATA3 (2.2.1.1.3)	0.995	0.661	0.677	0.267	0.149
PEAKS039665	ESR1 (2.1.1.2.1)	1.004	0.674	0.742	0.36	0.144
PEAKS033184	TAL1 (1.2.3.1.1)	0.991	0.653	0.793	0.446	0.536
PEAKS038038	PR (2.1.1.1.3)	48.883	0.827	0.792	0.625	0.868
PEAKS038673	SIX-1 (3.1.6.1.1)	40.463	0.356	0.909	0.885	0.296
PEAKS038812	ZFP-28 (2.3.3.0.192)	49.214	0.727	0.929	0.77	0.733
PEAKS040149	EHF (3.5.2.4.1)	49.914	0.397	0.53	0.501	0.579

92436 merged TFBRs were obtained after merging four individual datasets PEAKS039626. The pivotal frequencies f_1_ = 46452, f_2_ = 5797, f_3_ = 12012, and f_4_ = 28175 were used for computation of FPCM = 24.901. Obviously, this value essentially exceeded the threshold 2.0 (or 3.0) therefore we concluded that there is a large number of FPs among 46452 orphans. In other words, the majority of orphans were, in fact, falsely generated TFBRs. In this case, we were forced to discard orphans and obtain the following six Chapman’s estimates for calculation of FNCMs by using formula (19):

45996(GEM,MACS),46011(GEM,PICS),43591(GEM,SISSRs),

45609(MACS,PICS),45783(MACS,SISSRs),and47198(PICS,SISSRs).

The value 43591 was classified as an outlier when we used test for outlier detection. Therefore we excluded it and the final version of the expected number of genuine binding regions (N_2_^e^ = 46119) was computed by averaging five remained Chapman’s estimates. The resulted values of FPCM and FNCMs for PEAKS039626 meta-set are presented in Table 1. It is interesting to note that some high values of FPCM can be easily explained by abnormal outcome of one of the peak callers. Thus, GEM, MACS, PICS, and SISSRs generated 41827, 45318, 78011, and 43215 TFBRs correspondingly in case of PEAKS035099. It seems likely that PICS over-generated a large number of TFBRs and many of them were classified by FPCM as FPs.

Basically, FPCM recommends to remove or not the orphans while the FNCMs allow to select more reliable dataset from meta-set. Thus, FPCM recommended to remove orphans from PEAKS039626, PEAKS038673, PEAKS038812, and PEAKS040149, see FPCM values in Table 1. FNCM recommended to select MACS-generated datasets PEAKS035099, PEAKS039626, PEAKS033837, PEAKS039665, PEAKS033184, PEAKS038673, and PEAKS038812 while in cases of PEAKS038038 and PEAKS040149 FNCMs recommended to select GEM-generated datasets.

### Comprehensive quality control of ChIP-Seq datasets in the GTRD database

The first release of GTRD [28] has been prepared without taking into account the quality control of ChIP-Seq TFBR datasets. For the second release, we have computed the quality metrics FNCMs and FPCMs for all available datasets in GTRD according to the proposed algorithm. Then we studied the influence of presence/absence of input control in ChIP-Seq experiments on quality of TFBRs datasets generated by distinct peak callers. Four classification models: perceptron, Fisher’s discriminant model, logistic regression, and support vector machine (SVM)–were exploited to reveal putative relation between presence/absence of input control and our metrics FNCMs and FPCM. The strength of putative relation was measured by accuracy of the classification models, which was defined as the fraction of all correctly classified instances.

We applied the mentioned classification models to all 5084 human datasets in GTRD where input controls were available for 4033 (79.3%) datasets. To control the overfitting of the classification models we divided the whole set of datasets into equal-halves: training subset and test subset. Table 2 contains the computed values of accuracies of classification models. According to these values, one can conclude that there is a strong relation between presence/absence of input control and our metrics, FNCMs and FPCM, because each model correctly classified the majority (81.2%– 90.5%) of the tested subset. This conclusion is quite reliable because it is invariant with respect to the choice of the classification model, and the differences between accuracies observed on training and test sets are negligible.

**Table 2:** Accuracies of the classification models.

Classification model type	Training subset	Test subset
Perceptron	0.817	0.814
Fisher’s discriminant model	0.823	0.812
Logistic regression	0.869	0.861
SVM	0.918	0.905

**Fig 2:**
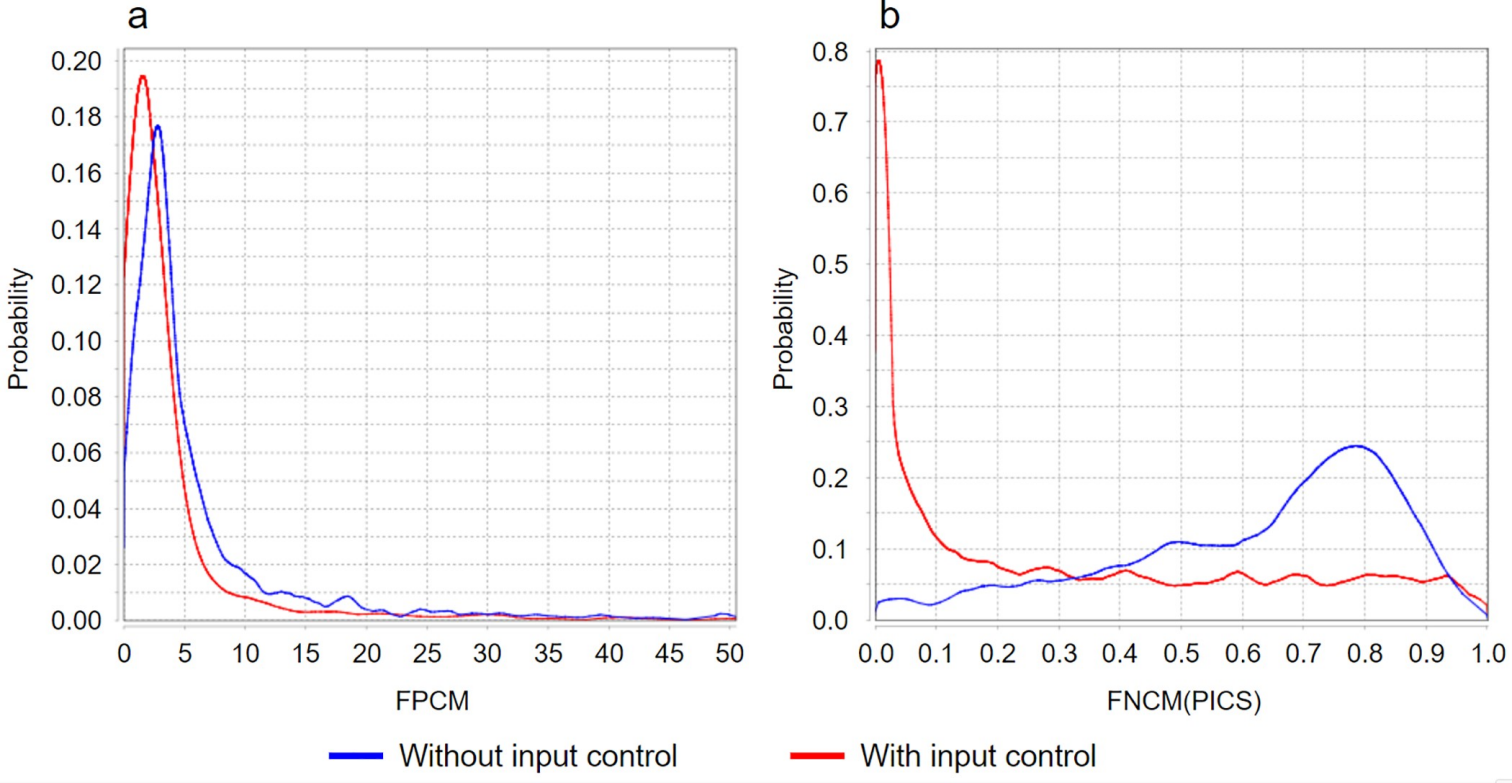
Empirical densities of (a) FPCM and (b) FNCM obtained for peak caller PICS.

To highlight the explicit form of the revealed relation, we calculated the mean values of FNCM and FPCM for datasets with and without input control separately. It is important to note that we calculated two versions of the FPCM average. For the first version, we used all available 5084 FPCM values, while for the second version we removed 132 (2.6%) FPCM values that exceeded value 100.0, because these huge values (such as 4079.4 or 12663.4) are abnormal and can result in misleading conclusions. Thus, if all 5078 FPCM values are used, then one can conclude that mean value of FPCM (namely, 19.918, see 1-st row of Table 3) for datasets with control is greater than the mean value of FPCM 11.876 for datasets without control. However, after removing 132 outliers (abnormal values) one can conclude that mean value of FPCM (namely, 3.923, see 2-nd row of Table 3) for datasets with control is less, than the mean value of FPCM 8.562 for datasets without control. Empirical densities of FPCM (see Fig 2(A)) confirmed correctness of the second version of FPCM average. On the base of this version and all FNCM averages in Table 3, we made a conclusion that the absence of input control resulted in increase of FP rate and in decrease of FN rates of MACS, PICS, and SISSRs. Using Wilcoxon rank sum test we have found that FPCM and almost all FNCMs (excluding FNCMs for MACS) made the statistically significant contribution into discrimination between presence/absence of input control, see the corresponding p-values in Table 3. Fig 2(B) demonstrates the densities of FNCM(PICS), which is the most significant feature for discrimination.

**Table 3:** Mean values of the quality control metrics FPCM and FNCMs calculated on 5078 human ChIP-Seq datasets available in the GTRD database.

Quality metrics	All datasets	Datasets with input control	Datasets without input control	Wilcoxon test (Z-score)	p-value
FPCM(first version)	18.251	19.918	11.876	16.482	< 10^−14^
FPCM(second version)	5.655	3.923	8.562	17.026	< 10^−14^
FNCM(GEM)	0.509	0.516	0.484	3.997	6.4 * 10^−5^
FNCM(MACS)	0.651	0.645	0.672	0.864	0.389
FNCM(PICS)	0.36	0.292	0.62	28.461	< 10^−14^
FNCM(SISSRs)	0.454	0.398	0.668	24.753	< 10^−14^

### Peak caller comparison

Despite the current existence of more than 30 published peak callers, various comparative analyses of them did not reveal the best one. These comparisons were performed frequently on a small number of datasets and distinct metrics were exploited for comparison. We also performed comparative analysis of GEM, MACS, SISSRs, and PICS by using 5084 human datasets and FNCMs. For this purpose, we determined the priority of peak callers in descending order of FNCMs for each dataset. Then we counted the frequencies of all 4! = 24 distinct priorities. Expected proportion of each priority was defined as its probability when all distinct priorities are equally probable, hence expected proportion is equal to 1 / 4! = 0.042. The following priority appeared to be the most frequent among distinct ones:

MACS>GEM>SISSRs>PICS.

On the one hand, the observed proportion 0.181 of this priority essentially and significantly (p-value < 10^−20^) exceeded the expected probability, because the ratio between observed and expected proportions (say, R_o/e_) was equal to 4.3. Statistical significance was estimated with the help of binomial distribution. Importantly, this excess was invariant with respect to the presence or absence of input control, see Table 4.

**Table 4:** The most frequent arrangements of the peak callers.

Priority	Type of datasets	Observed proportion	Ratio between observed and expected proportions, R_o/e_
MACS > GEM > SISSRs > PICS	All datasets	0.181	4.3
	Datasets with input control	0.195	4.6
	Datasets without input control	0.156	3.7
{MACS and GEM} > {SISSRS and PICS}	All datasets	0.469	5.6
	Datasets with input control	0.544	6.6
	Datasets without input control	0.338	4.1
{SISSRs, MACS and PICS} > GEM	Datasets without input control	0.635	2.5

On the other hand, we could not accept this priority as the general tendency among peak callers, because the majority (namely, 81.8%) of datasets had other priorities. To obtain more reliable inference, we considered the relaxed arrangements for comparison of pairs of peak callers. In this case the most frequent priority among 12 distinct ones was

{MACSandGEM}>{SISSRsandPICS}.

The observed proportion 0.469 of this priority also essentially (R_o/e_ = 5.6) and significantly (p-value < 10^−20^) exceeded the expected proportion 1 / 12 = 0.083, and about a half of datasets had this priority, see Table 4. Therefore, one can conclude that, in general, MACS and GEM outperformed SISSRs and PICS. It is important to note that the reliability of this conclusion is increasing during transition from all datasets to datasets with input control, because the observed proportion of the corresponding priority increased from 0.469 to 0.568. This conclusion is also confirmed by FNCM values contained in Table 3. Finally, it is interesting to note that GEM appeared to produce weaker results when the input control was absent, and the priority

{SISSRs,MACS,andPICS}>GEM

was observed for majority (63.5%) of datasets without input control. Tables 3 and 4 illustrate this conclusion.

### Relationships between the proposed quality metrics and other features of ChIP-Seq datasets

There are, at least, two types of ChIP-Seq dataset features in addition to the proposed quality metrics. The first type features are well-known standard quality metrics developed by the ENCODE consortium. For instance, metrics such as NRF, PBC1, PBC2, NSC, and RSC assess quality of read alignment to individual genomes. The second type features can be easily determined on the base of characteristics generated by individual peak callers. For example, MACS assigned such characteristics as ‘Fold enrichment’, ‘FDR’ (False Discovery Rate), ‘Tags number’ and ‘–lg(p-value)’ to each generated TFBR. To obtain second type features for the whole dataset we averaged available characteristics over generated TFBRs in given dataset.

**Fig 3:**
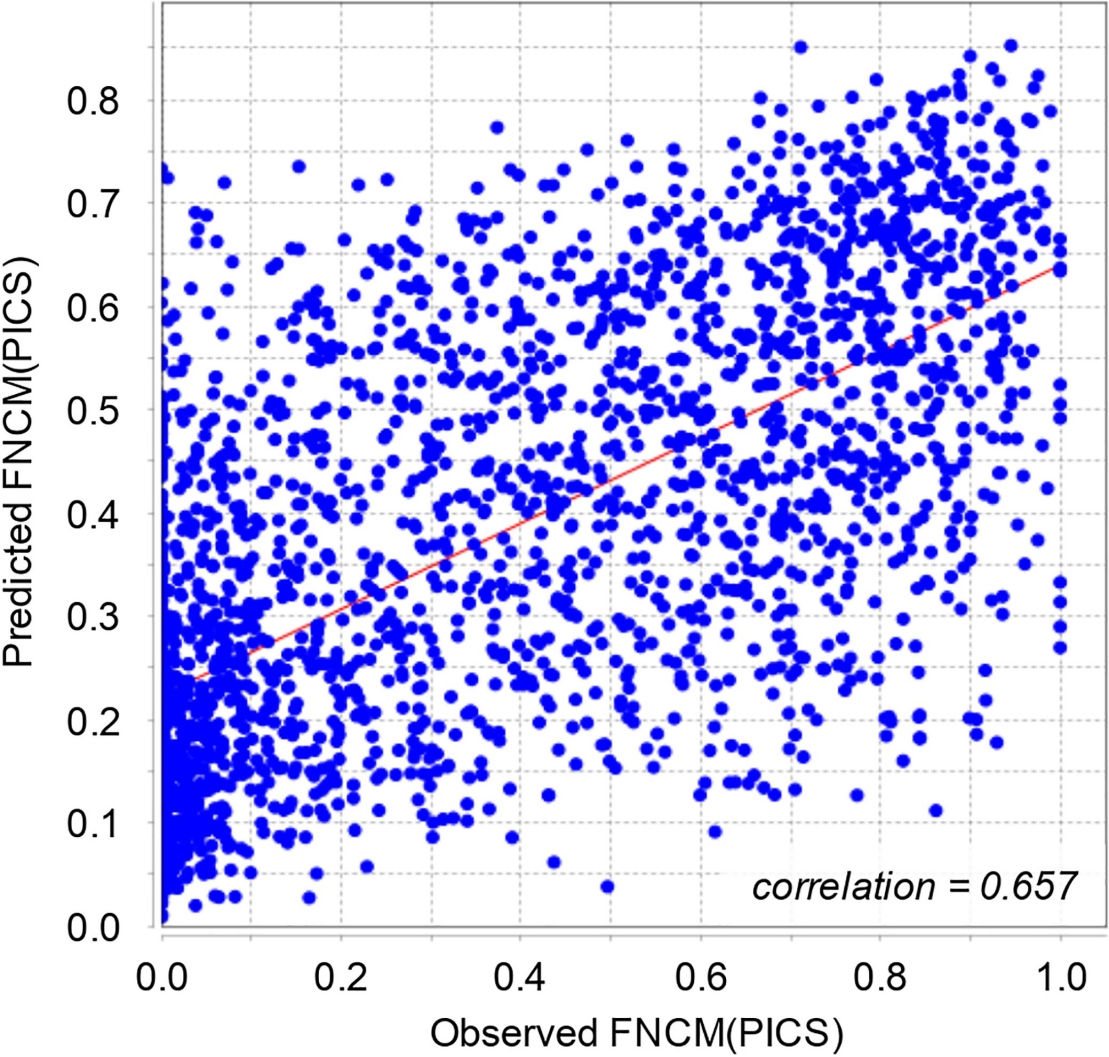
Relationship between FNCM(PICS) observed and predicted by the random forest regression model.

To study relations between the proposed metrics and the features of both types we performed regression analysis. For this purpose, we applied three multiple regression models, namely, ordinary least squares (OLS), random forest (RF), and SVM to 5084 human datasets in GTRD. The strength of relationships between the features and the FNCM/FPCM metrics was measured by application of Pearson’s correlation between observed and predicted values of the metrics. To avoid the impact of overfitting the regression models we divided the whole set of 5084 datasets into the following equal-sizes subsets: training and test subsets. The regression models were fitted to the training subset while the correlation between observed and predicted values were calculated on the test subset. The maximal values of correlation 0.657 and 0.644 were achieved by RF models, see Table 5. In first case, regression model described the relation between FNCM (PICS) and the ENCODE quality metrics while the second regression described the relation between FNCM (GEM) and the peak caller characteristics. In general, moderate values of correlations indicate that there are no strong relations between the proposed metrics and the existing features, in particular, see Fig 3. In other words, there are no combinations of known features that can replace FNCM or FPCM.

**Table 5:** Relationships between the proposed quality metrics and features of both types.

Features type	Quality metric	Regression model	Correlation between observed and predicted quality metrics	Relevant feature	Individual correlation between quality metric and relevant feature
Quality metrics introduced by ENCODE	FNCM (GEM)	OLS RF SVM	0.472 0.611 0.545	FRiP(GEM) PBC1 NRF	0.302 0.301 0.293
	FNCM (MACS)	OLS RF SVM	0.336 0.413 0.327	NRF PBC1	0.279 0.275
	FNCM (PICS)	OLS RF SVM	0.415 0.657 0.475	FRiP(PICS)	0.392
	FNCM (SISSRs)	OLS RF SVM	0.259 0.451 0.295	FRiP(SISSRs)	0.157
	FPCM	OLS RF SVM	0.044 0.104 0.064	-	-
Peak caller characteristics	FNCM (GEM)	OLS RF SVM	0.233 0.644 0.596	Noise -lg(p-value) -lg(q-value)	-0.233 0.181 0.187
	FNCM (MACS)	OLS RF SVM	0.371 0.578 0.512	Tags number -lg(p-value)	-0.172 0.256
	FNCM (PICS)	OLS RF SVM	0.031 0.037 0.509	Score	-0.267
	FNCM (SISSRs)	OLS RF SVM	0.353 0.471 0.442	-lg(p-value) Fold enrichment Tags number	0.355 -0.226 -0.187
	FPCM	OLS RF SVM	0.119 0.464 0.287	-	-

Despite the absence of strong relations, it is fruitful to interpret the moderate associations between the individual features and FNCM or FPCM. For this purpose, for each FNCM and FPCM we determined the relevant features as features with absolute values of individual correlations between them and proposed metrics greater than 0.1. All revealed relevant features representing the quality metrics from ENCODE had positive correlations with FNCMs, see Table 5. In other words, the more qualitative dataset from the point of view of the ENCODE metrics, the lower FN rates from the point of view of FNCMs. Thus, there is positive association between the ENCODE metrics and FNCMs.

When we performed analogous comparison between FNCMs and the peak caller characteristics, it appeared that, on the one hand, there existed positive association between FNCMs and the probabilistic characteristics such as–lg(p-value) or–lg(q-value) and, on the other hand, there was the surprising negative association between FNCMs and the not-probabilistic characteristics such as Fold enrichment and Tags number.

**Fig 4:**
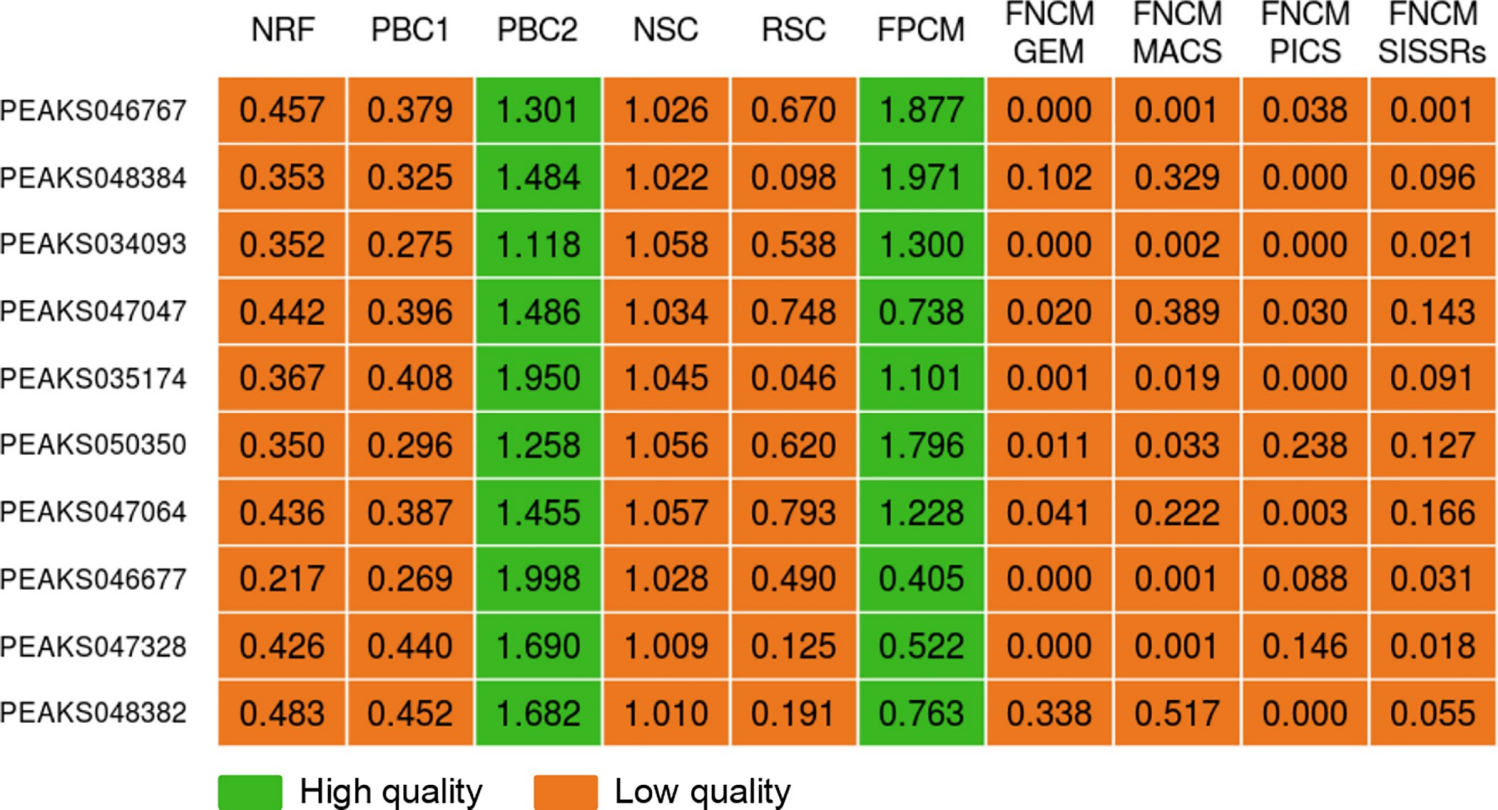
Quality metrics values for some low-quality ChIP-Seq data from GTRD.

Finally, it was important to demonstrate the usefulness of the proposed quality metrics, when almost all ENCODE control metrics failed. Thus, Fig 4 demonstrates such cases. On the one hand, such characteristics as NRF, PBC1, NSC and RSC recommended to exclude these data from further analysis. On the other hand, in almost all cases FNCM indicated the high rate of FNs. In other words, peak callers overlooked numerous genuine TFBRs. However, FPCM indicated the low rate of FPs, therefore it recommended to use whole merged datasets without removing orphans for applications such as identification of TFBSs within ChIP-Seq datasets, or comparison of motif prediction methods.

### Identification of TFBSs within ChIP-Seq datasets

Accurate identification of TFBSs (or site motifs) is still the big challenge in bioinformatics. Though comprehensive study of abilities of the existing models for motif prediction is beyond of our study, we demonstrate below that reasonable application of FPCM can essentially improve the accuracy of TFBS identification within TFBRs. To confirm this, we applied two PWM models (namely, MATCH and HOCOMOCO) to some datasets of TFBRs when these models shared the same matrices.

**Fig 5:**
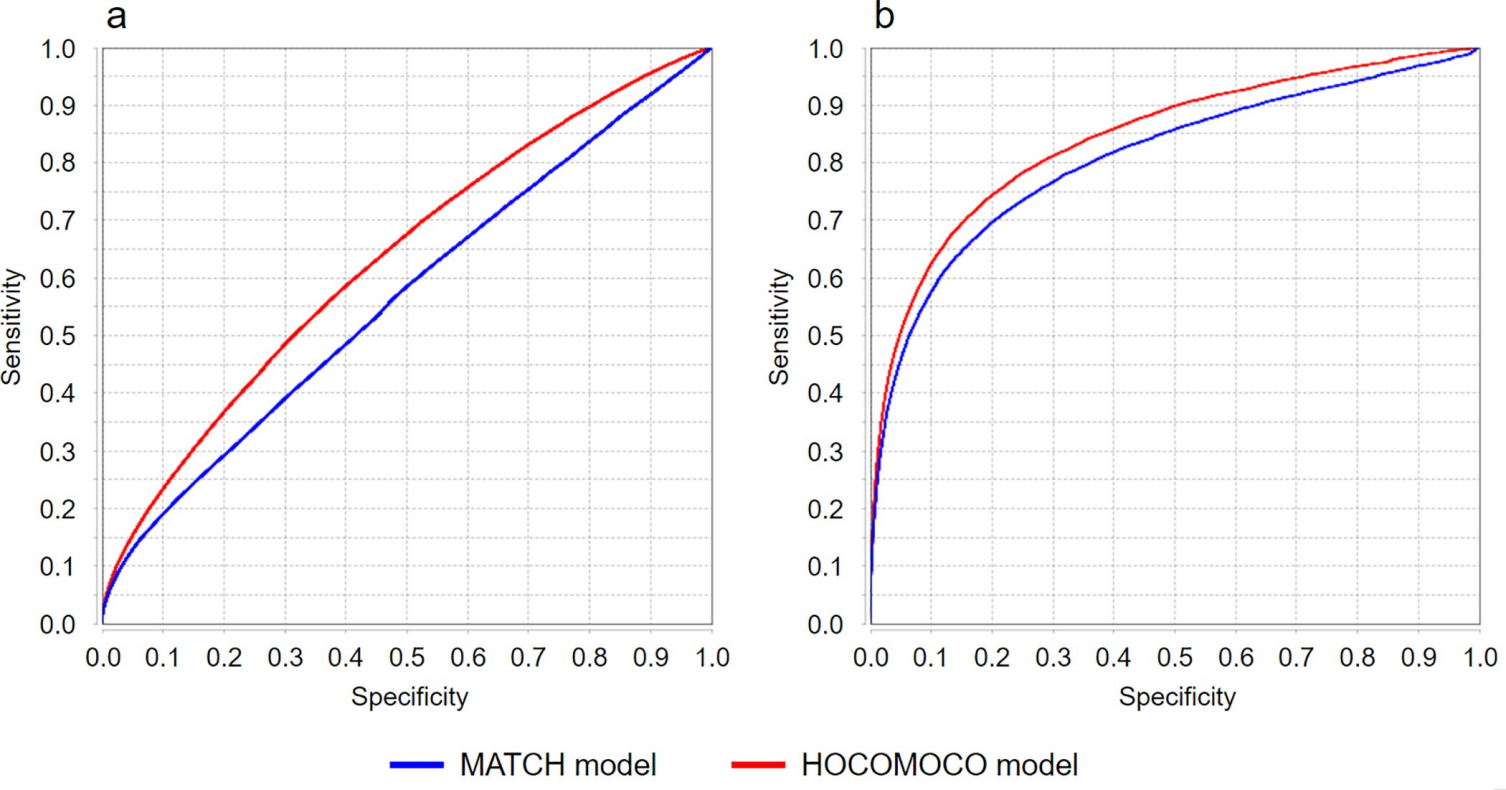
ROC curves for (a) whole dataset PEAKS038038 and (b) for PEAKS038038 without orphans.

In general, accuracy of TFBS identification within a given dataset of TFBRs depends on, at least, four following factors: 1) quality of matrix, 2) quality of scoring method, 3) quality of dataset, and 4) unknown proportion of tethered binding when a given TF bound to DNA fragment not because it recognized its site, but due to protein-protein interaction with another TF that, in turn, bounds to DNA directly. To demonstrate the influence of dataset quality on motif identification, we built ROC curves and calculated AUCs on datasets of TFBSs mentioned in Table 1. In particular, Fig 5 contains the ROC curves obtained on the whole dataset PEAKS038038 and the one without orphans. On the base of low values of AUCs (0.633 for the HOCOMOCO model and 0.565 for the MATCH model, see Table 6) one can conclude that both models had low predictive abilities. However, according to Table 1, FPCM was equal to 48.883, hence majority of orphans in PEAKS038038 were TFBRs falsely identified by peak callers. After removing orphans, the values of AUCs (0.843 for the HOCOMOCO model and 0.808 for the MATCH model, see Table 6) increased essentially. The analogous effect of significant increase of AUCs after removing orphans has been observed for other datasets (such as PEAKS039626, PEAKS038673, PEAKS038812, and PEAKS040149). According to Table 1, the FPCM values calculated for these datasets considerably exceeded 1.0. However, exclusion of orphans did not lead to essential increase of the AUC values for such datasets as PEAKS035099, PEAKS033754, PEAKS033837, PEAKS039665, and PEAKS033184. This effect was not unexpected because the corresponding FPCM values for these datasets were close to 1.0.

**Table 6:** Values of area under ROC curve for datasets mentioned in Table 1.

Dataset	Whole dataset		Without orphans	
	MATCH	HOCOMOCO	MATCH	HOCOMOCO
PEAKS035099	0.880	0.888	0.887	0.896
PEAKS039626	0.684	0.691	0.849	0.858
PEAKS033754	0.780	0.794	0.783	0.795
PEAKS033837	0.620	0.655	0.628	0.663
PEAKS039665	0.778	0.817	0.786	0.825
PEAKS033184	0.790	0.824	0.840	0.868
PEAKS038038	0.565	0.633	0.808	0.843
PEAKS038673	0.564	0.556	0.813	0.844
PEAKS038812	0.603	0.640	0.776	0.796
PEAKS040149	0.595	0.578	0.722	0.623

It is interesting to note that the HOCOMOCO model outperformed the MATCH model. This outperformance can be the result of taking into account the background nucleotide composition of the motifs by the HOCOMOCO model (unlike the MATCH model) [29]. However, this superiority was not high because of small differences between corresponding AUCs. Anyway, the differences between AUCs indicated that distinctions between the PWM models were less considerable than effect of appropriate removing orphans.

Finally, the same relations between removing orphans and essential increase of AUCs have also been observed when we merged datasets of TFBRs obtained by application of a single peak caller to the distinct ChIP-Seq sets of reads when the same TF was studied in different ChIP-Seq experiments. In particular, Table 7 contains AUCs obtained for the following TFs: ATF-1, SRF and NF-E2. We selected these TFs because the corresponding values of FPCM exceeded threshold 2.0, hence FPCM actually recommended to remove orphans.

**Table 7:** Values of area under ROC curve when peaks from distinct ChIP-Seq studies were merged.

TF name (TF-class)	Dataset type	GEM		MACS		PICS		SISSRs	
		HOCOMOCO	MATCH	HOCOMOCO	MATCH	HOCOMOCO	MATCH	HOCOMOCO	MATCH
ATF-1 (1.1.7.1.2)	whole	0.57	0.57	0.51	0.49	0.52	0.51	0.53	0.52
	without orphans	0.78	0.78	0.61	0.6	0.91	0.9	0.84	0.83
SRF (5.1.2.0.1)	whole	0.64	0.61	0.57	0.56	0.57	0.55	0.57	0.56
	without orphans	0.77	0.74	0.65	0.63	0.81	0.8	0.82	0.81
NF-E2 (1.1.1.2.1)	whole	0.7	0.69	0.67	0.66	0.59	0.58	0.61	0.6
	without orphans	0.78	0.76	0.76	0.75	0.74	0.73	0.89	0.88

### Implementation

The developed algorithm for determination of FPCM and FNCM for ChIP-Seq datasets was implemented as a plugin for the BioUML platform [30]: https://ict.biouml.org/bioumlweb/chipseq_analysis.html. BioUML is an open source comprehensive bioinformatics platform, free for non-commercial use.

## Conclusions

In this study we developed two novel metrics: FPCM and FNCM, which allow to control FP and FN rates of peak callers for assessment of quality of TFBR datasets. The main aim of the developed metrics is the selection of the most reliable datasets or recommendation of dataset modification by removing the orphans.

After estimation of FNCM and FPCM metrics for all human ChIP-Seq datasets from GTRD, we observed strong relations between presence/absence of input control in ChIP-Seq experiment and the FNCM and FPCM metrics. In particular, the absence of input control resulted in increase of FP rate and decrease of FN rates of the peak callers MACS, PICS, and SISSRs. In addition, we performed a comparative analysis of four peak callers: MACS, PICS, GEM and SISSRs using FNCM metrics. It was revealed that, in general, MACS and GEM outperformed SISSRs and PICS, especially when input controls were available for ChIP-Seq datasets. Moreover, comparative analysis of the existing quality metrics developed by the ENCODE project, FNCM and FPCM metrics and characteristics generated by individual peak callers has been performed. No strong relationships between FNCM and FPCM metrics and existing quality metrics or peak callers’ characteristics have been revealed. In other words, there are no combinations of known metrics and peak callers’ characteristics that can replace FNCMs and FPCM metrics. Thus, reasonable application of FPCM can considerably improve the accuracy of TFBS identification within ChIP-Seq datasets.

## References

[1] ENCODE Project Consortium. An integrated encyclopedia of DNA elements in the human genome. Nature. 2012 9 6;489:57–74. 10.1038/nature11247 22955616 PMC3439153

[2] Yevshin I, Sharipov R, Kolmykov S, Kondrakhin Y, Kolpakov F. GTRD: a database on gene transcription regulation-2019 update. Nucleic Acids Res. 2019 1;47(D1):D100–D105. 10.1093/nar/gky1128 30445619 PMC6323985

[3] Oki S, Ohta T, Shioi G, Hatanaka H, Ogasawara O, Okuda Y, et al ChIP-Atlas: a data-mining suite powered by full integration of public ChIP-seq data. EMBO reports. 2018 11 9;19(12):e46255 10.15252/embr.201846255 30413482 PMC6280645

[4] Cheneby J, Gheorghe M, Artufel M, Mathelier A, Ballester B. ReMap 2018: an updated atlas of regulatory regions from an integrative analysis of DNA-binding ChIP-seq experiments. Nucleic Acids Res. 2018 1 4;46(D1):D267–D275. 10.1093/nar/gkx1092 29126285 PMC5753247

[5] Landt SG, Marinov GK, Kundaje A, Kheradpour P, Pauli F, Batzoglou S, et al ChIP-seq guidelines and practices of the ENCODE and modENCODE consortia. Genome Res. 2012;22(9):1813–1831. 10.1101/gr.136184.111 22955991 PMC3431496

[6] Chao A, Bunge J. Estimating the number of species in a stochastics abundance model. Biometrics. 2002 9;58:531–539. 12229987 10.1111/j.0006-341x.2002.00531.x

[7] Woodward M. Epidemiology: Study Design and Data Analysis. London: Chapman and Hall/CRC; 2013.

[8] Hope VD, Hickman M, Tilling K. Capturing crack cocaine use: estimating the prevalence of crack cocaine use in London using capture–recapture with covariates. Addiction. 2005 Sep 15;100(11):1701–1708. 10.1111/j.1360-0443.2005.01244.x 16277630

[9] Kel AE, Gossling E, Reuter I, Cheremushkin E, Kel-Margoulis OV, Wingender E. MATCHTM: a tool for searching transcription factor binding sites in DNA sequences. Nucleic Acids Res. 2003 7 1;31(13):3576–3579. 10.1093/nar/gkg585 12824369 PMC169193

[10] Bailey TL, Johnson J, Grant CE, Noble WS. The MEME Suite. Nucleic Acids Res. 2015 7 1;43(W1):W39–W49. 10.1093/nar/gkv416 25953851 PMC4489269

[11] Kulakovskiy IV, Vorontsov IE, Yevshin IS, Soboleva AV, Kasianov AS, Ashoor H, et al HOCOMOCO: expansion and enhancement of the collection of transcription factor binding sites models. Nucleic Acids Res. 2016 1 4;44(D1):D116–D125. 10.1093/nar/gkv1249 26586801 PMC4702883

[12] Khan A, Fornes O, Stigliani A, Gheorghe M, Castro-Mondragon JA, van der Lee R, et al JASPAR 2018: update of the open-access database of transcription factor binding profiles and its web framework. Nucleic Acids Res. 2018 1 4;46(D1), D260–D266. 10.1093/nar/gkx1126 29140473 PMC5753243

[13] Hume MA, Barrera LA, Gisselbrecht SS, Bulyk ML. UniPROBE, update 2015: new tools and content for the online database of protein-binding microarray data on protein-DNA interactions. Nucleic Acids Res. 2015 1 28;43(D1):D117–D122. 25378322 10.1093/nar/gku1045 PMC4383892

[14] Thomas R, Thomas S, Holloway AK, Pollard KS. Features that define the best ChIP-Seq peak calling algorithms. Brief Bioinform. 2017 5;18(3):441–450. 10.1093/bib/bbw035 27169896 PMC5429005

[15] Laajala TD, Raghav S, Tuomela S, Lahesmaa R, Aittokallio T, Elo LL. A practical comparison of methods for detecting transcription factor binding sites in ChIP-seq experiments. BMC Genomics. 2009 12 18;10(1):618. 20017957 10.1186/1471-2164-10-618 PMC2804666

[16] Harmanci A, Rozowsky J, Gerstein M. MUSIC: identification of enriched regions in Chip-Seq experiments using a mappability-corrected multiscale signal processing framework. Genome Biol. 2014 10 8;15(10):474 10.1186/s13059-014-0474-3 25292436 PMC4234855

[17] Koohy H, Down TA, Spivakov M, Hubbard T. A comparison of peak callers used for DNase-Seq data. PLoS ONE. 2014 5 8;9(5):e96303 10.1371/journal.pone.0096303 24810143 PMC4014496

[18] Micsinai M, Parisi F, Strino F, Asp P, Dynlacht BD, Kluger Y. Picking ChIP-seq peak detectors for analyzing chromatin modification experiments, Nucleic Acids Res. 2012 5 1;40(9):e70 10.1093/nar/gks048 22307239 PMC3351193

[19] Guo Y, Mahony S, Gifford DK. High resolution genome wide binding event finding and motif discovery reveals transcription factor spatial binding constraints. PLoS Comput. Biol. 2012 8 9;8(8):e1002638 10.1371/journal.pcbi.1002638 22912568 PMC3415389

[20] Zhang Y, Liu T, Meyer CA, Eeckhoute J, Johnson DS, Bernstein BE, et al Model-based analysis of ChIP-Seq (MACS). Genome Biol. 2008 9 17;9(9):R137 10.1186/gb-2008-9-9-r137 18798982 PMC2592715

[21] Zhang X, Robertson G, Krzywinski M, Ning K, Droit A, Jones S, et al PICS: probabilistic inference for ChIP-seq. Biometrics. 2011 3 14;67(1):151–163. 10.1111/j.1541-0420.2010.01441.x 20528864

[22] Narlikar L, Jothi R. ChIP-Seq data analysis: identification of protein-DNA binding sites with SISSRs peak-finder. Methods Mol. Biol. 2011 11 18;802:305–322. 10.1007/978-1-61779-400-1_20 PMC4783134 22130889

[23] Chao A. Estimating the population size for capture–recapture data with unequal catchability. Biometrics. 1987 12;43(4):783–791. 3427163

[24] Lanumteang K, Bohning D. An extension of Chao’s estimator of population size based on the first three capture frequency counts. Comput. Stat. Data An. 2011 2 22;55(7):2302–2311.

[25] Zelterman D. Robust estimation in truncated discrete distributions with application to capture-recapture experiments. J. Stat. Plan. Inf. 1988 3 25;18(2):225–237.

[26] McCrea RS, Morgan BJT. Analysis of Capture-Recapture Data. London: Chapman and Hall/CRC; 2014.

[27] Chapman DH. Some properties of the hypergeometric distribution with applications to zoological surveys. Univ. Calif. Publ. Stat. 1951;1:131–160.

[28] Yevshin I, Sharipov R, Valeev T, Kel A, Kolpakov F. GTRD: a database of transcription factor binding sites identified by ChIP-seq experiments. Nucleic Acids Res. 2017 1;45(D1):D61–D67 10.1093/nar/gkw951 27924024 PMC5210645

[29] Kulakovskiy IV, Boeva VA, Favorov AV, Makeev VJ. Deep and wide digging for binding motifs in ChIP-Seq data. Bioinformatics. 2010 10 15;26(20):2622–3. 10.1093/bioinformatics/btq488 20736340

[30] Kolpakov F, Akberdin I, Kashapov T, Kolmykov S, Kondrakhin Y, Kutumova E, et al BioUML: an integrated environment for systems biology and collaborative analysis of biomedical data. Nucleic Acids Res [Preprint]. 2019 5 27 Available from: https://academic.oup.com/nar/advance-article/doi/10.1093/nar/gkz440/5498754 10.1093/nar/gkz440. PMC6602424 31131402

